# A Phase I Study of Locoregional High-Dose Autologous Natural Killer Cell Therapy With Hepatic Arterial Infusion Chemotherapy in Patients With Locally Advanced Hepatocellular Carcinoma

**DOI:** 10.3389/fimmu.2022.879452

**Published:** 2022-06-02

**Authors:** Woo Kyun Bae, Byung Chan Lee, Hyeon-Jong Kim, Je-Jung Lee, Ik-Joo Chung, Sung Bum Cho, Yang Seok Koh

**Affiliations:** ^1^ Department of Hematology-Oncology, Chonnam National University Medical School and Chonnam National University Hwasun Hospital, Hwasun, South Korea; ^2^ Immunotherapy Innovation Center, Chonnam National University Medical School and Chonnam National University Hwasun Hospital, Hwasun, South Korea; ^3^ Department of Radiology, Chonnam National University Medical School and Chonnam National University Hwasun Hospital, Hwasun, South Korea; ^4^ Vaxcell-Bio Therapeutics, Hwasun, South Korea; ^5^ Department of Gastroenterology, Chonnam National University Medical School and Chonnam National University Hwasun Hospital, Hwasun, South Korea; ^6^ Department of General Surgery, Chonnam National University Medical School and Chonnam National University Hwasun Hospital, Hwasun, South Korea

**Keywords:** hepatocellular carcinoma, natural killer (NK) cell, hepatic arterial infusion chemotherapy (HAIC), clinical trial, autologous

## Abstract

**Background:**

To explore the feasibility and safety of natural killer (NK) cell therapy in HCC, we performed a prospective, open-label, phase I trial to evaluate the synergistic effect of locoregional high-dose autologous NK cell therapy in combination with hepatic arterial infusion chemotherapy (HAIC).

**Methods:**

Patients with locally advanced HCC who were refractory to the standard treatment were eligible for this study. Patients received expanded and activated NK cells for 5 consecutive days in a dose-escalating manner (dose 2.5×10^8^, 5×10^8^, 10×10^8^ NK cells/injection) through hepatic arterial infusion following 4 cycles of HAIC with 5-fluorouracil (750 mg/m^2^) and cisplatin (25 mg/m^2^). The primary endpoint was the safety of NK cell-based immunotherapy, and the secondary endpoints were objective response rate (ORR), progression-free survival (PFS), overall survival (OS), and immunologic responses.

**Results:**

Of the 11 patients enrolled, the confirmed ORR was 63.6% (complete response [CR]: 36.4%, confirmed partial response [PR]: 27.3%). Stable disease (SD) and progressive disease (PD) were observed in two patients (18.2%) each, resulting in a disease control rate (DCR) of 81.8%. The median PFS and OS were 10.3 and 41.6 months, respectively. There were no incidences of decompensation or severe adverse events during HAIC, and no adverse events related to NK cell infusion were noted.

**Conclusion:**

The combination of HAIC and locoregional high-dose NK cell therapy is a safe and effective treatment for locally advanced HCC patients who were refractory to the standard treatment. This result warrants further development of this novel treatment to establish its efficacy in HCC.

**Clinical Trial Registration:**

cris.nih.go.kr, identifier KCT0003973.

## Introduction

Hepatocellular carcinoma (HCC) is currently the sixth most common cancer and the third leading cause of cancer-related mortality worldwide in 2020, and the treatment strategies for HCC are selected based on tumor staging (1). Although the Barcelona Clinic Liver Cancer (BCLC) guideline has been widely accepted in clinical practice, there have been regional differences in the treatment of HCC (2). This is especially true in patients with portal vein thrombosis without extrahepatic metastasis, wherein prognosis remains poor. Systemic treatment with sorafenib or lenvatinib has been a useful therapeutic approach for HCC; however, its effect on the survival outcome has been limited with the median OS of 10.7 and 7.9 months in HCC patients treated with sorafenib and placebo, respectively (3). Therefore, there still remains a significant need for new, active treatments in locally advanced HCC.

NK cells are the essential components of the innate immune system in the liver, accounting for 30%–50% of the intrahepatic lymphocytes. Studies have reported that the number and function of NK cells were significantly reduced in HCC patients, and the reduction of tumor-infiltrating NK cells was associated with poor survival in the advanced stages of HCC (4, 5). This implicates the important role of intrahepatic NK cells in the immune surveillance against HCC. Thus, various approaches have been utilized to overcome NK cell dysfunction and to restore NK cell activity in the immune defense against HCC (6).

In Korea and Japan, hepatic arterial infusion chemotherapy (HAIC) has been applied for the treatment of locally advanced HCC. Theoretically, HAIC has several advantages, including higher efficacy and lower systemic toxicity than systemic therapy, as the infusion of drugs through the hepatic artery provides direct delivery of chemotherapeutic agents to the tumor cells. 5-fluorouracil (5-FU) and cisplatin are the most common regimen for the hepatic arterial infusion. Notably, it has been reported that some chemotherapeutic agents including 5-FU and cisplatin have immunomodulatory effects as a potent inducer of NK cell activity (7, 8). Given the predictive role, we assumed that prior treatment with HAIC of 5-FU and cisplatin would enhance the cytotoxic effect of highly activated NK cells expanded ex vivo together with their direct antitumor activity on tumor cells.

On the basis of this hypothesis, we designed a phase 1 study in which 5-FU and cisplatin were administered alone through the hepatic arterial infusion to induce the tumor growth inhibition prior to NK cell infusion. In the current study, we aimed to evaluate the safety and efficacy of locoregional high-dose autologous NK cells (VAX-NK/HCC) generated by our novel system in combination with HAIC of 5-FU and cisplatin in patients who were not suitable for or refractory to the standard treatment.

## Patients and Methods

### Study Design and Treatment

**Figure 1:**
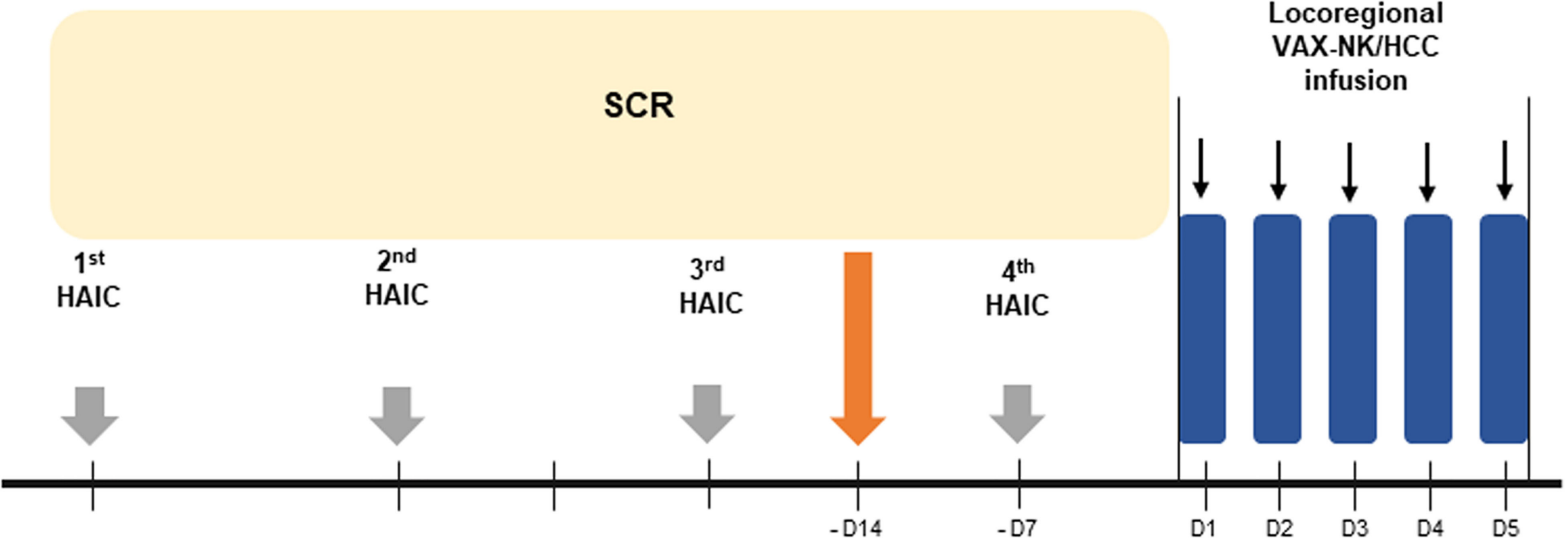
The schematic scheme for the treatment schedule of VAX-NK/HCC infusion following HAIC treatment. HAIC, hepatic arterial infusion chemotherapy; NK, natural killer; SCR, screening.

This study was a non-randomized, open-label, phase I trial with the dose escalation of VAX-NK/HCC cells in patients with advanced HCC. The primary endpoint of this study was to evaluate the safety of VAX-NK/HCC and HAIC combination treatment. The secondary endpoints were to evaluate ORR, PFS, OS, and immunologic response. The schematic diagram for the treatment schedule is summarized in **Figure 1**. In this study, a total of 4 cycles of HAIC were administered, with 750 mg/m^2^ of 5-FU and 25 mg/m^2^ of cisplatin for 4 days every 4 weeks (Q4W). For patients who were 65 years or older, had a history of grade 3 adverse events in the previous HAIC schedule, or had an estimated glomerular filtration rate of < 50 mL/min, a reduced dose of HAIC was administered, with 500 mg/m^2^ of 5-FU and 15 mg/m^2^ of cisplatin. Meanwhile, patients who achieved sustained SD or better based on the modified Response Evaluation Criteria in Solid Tumors (mRECIST) criteria after 2^nd^ cycle of HAIC were enrolled to receive VAX-NK/HCC. These selected patients underwent leukapheresis after the 3^rd^ HAIC cycle. This leukapheresis which was aimed at obtaining NK cells at this time was intended not to affect the chemotherapy schedule at all. VAX-NK/HCC was locoregionally administered for 5 consecutive days (high-dose NK cell therapy) while maintained in a fresh culture following the 4^th^ HAIC cycle. After VAX-NK/HCC administration, all adverse events were observed, and the next dose escalation was determined. Three patients each were treated with 2.5 × 10^8^ and 5.0 × 10^8^ cells. After the higher dose was determined as a tolerable dose, an additional five patients were enrolled and received a dose of 10 × 10^8^ cells.

### Patient Eligibility and Ethical Approval

Patients with intermediate and/or locally advanced HCC histologically confirmed by biopsy or typical radiological findings were eligible to participate in this study. All patients were not suitable for or failed curative treatments such as surgical resection, local ablation therapy, transarterial chemoembolization (TACE), or sorafenib. The inclusion criteria were as follows: age >18 years; an Eastern Cooperative Oncology Group performance status (ECOG PS) of 0 or 1; Child-Pugh liver function class A or B (9); macrovascular invasion; adequate hematologic functions (defined as neutrophils ≥ 1,500/uL, hemoglobin ≥ 9.0 g/dL, and platelet counts ≥ 75,000/uL); and adequate liver and renal functions (total bilirubin ≤ 2 mg/dL, alanine aminotransferase and aspartate aminotransferase ≤ 5 X the upper limit of the normal range [ULN], and serum creatinine ≤ 1.5 X ULN). Patients were excluded if they had received the immune cell-based therapy within 6 months from enrollment, liver transplantation, a history of a malignancy other than HCC within the last 5 years, and hypersensitivity to 5-FU or cisplatin.

All patients provided a written informed consent before participation, and all procedures associated with this study were conducted in accordance with Good Clinical Practice and the Declaration of Helsinki. This study was reviewed and approved by the Institutional Research Board of the Chonnam National University Hwasun Hospital (IRB No. CNUHH-2016-022).

### VAX-NK/HCC Generation and Quality Control

After performing leukapheresis to the patients, peripheral blood mononuclear cells (PBMCs) were isolated by density gradient centrifugation on lymphoprepTM (Axis-Shield, Oslo, Norway) solution. PBMCs were expanded with 100 Gy gamma-irradiated K562 cells with RPMI1640 medium supplemented with 10% heat-inactivated fetal bovine serum (FBS) and 4 mM/L L-glutamine (all from Gibco, Thermo Fisher Scientific, Waltham, MA, USA) in the presence of 10 U/mL recombinant human IL-2 (Peprotech, Rocky Hill, NJ, USA), and the medium and cytokine were replaced by every 2-3 days. After 7 days of culture, the concentration of IL-2 was increased to 100 U/mL, and 10 U/mL recombinant human IL-15 (Peprotech) was also added to the medium. On day 14 to 18 of culture, NK cells were harvested and resuspended in 500 mL Hartman solution (JW Pharmaceutical, Seoul, Korea) with 0.4% human serum albumin (GC Pharma, Yongin, Korea).

All generated NK cells underwent quality and safety tests evaluated according to the standard operating protocols and test guidelines of Vaxcell-Bio Therapeutics, approved by the Korea Ministry of Food and Drug Safety. The purity of NK cells and expression for surface receptors were determined by flow cytometry after staining with the following monoclonal antibodies: FITC-conjugated anti-CD3, PE-cy5-conjugated anti-CD56, or PE-conjugated anti-CD16, anti-CD69, anti-CD94, and anti-NKG2D (all from BD Biosciences, Franklin Lakes, NJ, USA). To estimate cytotoxicity, the NK cells were stained with 0.05 μM Calcein-AM (Thermo Fisher Scientific, Invitrogen, USA) and cultured with K562 cells for 4 h at 4:1 effector to target (E:T) ratio. Then, propidium iodide (Gibco) was added and cells were analyzed on flow cytometry. Test for sterility, mycoplasma, and absence of virus were performed at 3 days before first harvest and another sterility test was carried out on day of final harvest. Gram stain and endotoxin were performed on day 14 to 18 of NK cell harvest.

### Assessment of Safety, Clinical Response, and Immunologic Response After VAX-NK/HCC Infusion

The adverse events (AEs), vital signs, and physical examination were evaluated. Safety was assessed and graded according to the National Cancer Institute Common Toxicity Criteria for Adverse Event (NCI-CTCAE v4.0). The tumor response was determined based on the mRECIST (10, 11). Radiologic imaging was performed at baseline within 5 weeks before the initiation of VAX-NK/HCC infusion by a computed tomography (CT) or magnetic resonance imaging (MRI) scan. Imaging scans were repeated 4 and 8 weeks after the treatment for tumor response evaluation. For immunologic responses, PBMCs and blood serum were collected from patients prior to VAX-NK/HCC infusion and at 1 week, 2 weeks, 4 weeks, and 8 weeks after VAX-NK/HCC infusion. Cytotoxicity was assessed based on a previously described method using Calcein-AM (Thermo Fisher Scientific) stained PBMCs and propidium iodide (Gibco) stained K562 cells at a 10:1 E:T ratio. IFN-γ, IL-10, and TGF-β levels were then measured in blood serum using enzyme-linked immunosorbent assay. Furthermore, the PBMC population, such as NK cells and T cells, was determined by flow cytometry analysis using the following monoclonal antibodies: FITC-conjugated anti-CD3, PE-cy5-conjugated anti-CD56, APC-conjugated anti-CD4, and PE-conjugated anti-CD8 (all from BD Biosciences). Meanwhile, the phenotype test for NK cell activation and inhibitory receptors were analyzed by flow cytometry using the following monoclonal antibodies: FITC-conjugated anti-CD3, PE-cy5-conjugated anti-CD56, PE-conjugated anti-CD16, anti-CD69, anti-CD94, anti-NKG2D, anti-NKp30, NKp44, NKp46, and CD158b (all from BD Biosciences).

### Hepatic Arterial Infusion Chemo-Port Implantation Technique

All hepatic arterial infusion chemo-port system (Celsite ST201C; B. Braun, Chasseneuil, France) implants were accessed through the common femoral artery under local anesthesia with 1% lidocaine, and a chemo-port chamber was implanted into the subcutaneous fat layer of the upper thigh below the groin. Vascular access was achieved with the Seldinger technique using a 4-French (4-Fr) micro-puncture set (MAK; Merit Medical, South Jordan, UT, USA). After the insertion of the 5-Fr introducer sheath (Radiofocus Introducer II; Terumo, Tokyo, Japan) into the superior mesenteric artery, celiac axis angiography was performed to confirm vascular anatomy and anatomical variations using a 5-Fr catheter (Yashiro catheter; Terumo, Tokyo, Japan). The gastroduodenal and right gastric arteries were then selected using a micro-catheter (Renegade STC 18; Boston Scientific, Marlborough, MA, USA) and 0.016-inch wire (ASAHI Meister; Asahi Intecc, Seto, Japan) and were routinely embolized with detachable micro-coils (Concerto detachable coils; Medtronic, Dublin, Ireland, or Interlock coils; Boston Scientific, Marlborough, MA, USA) to prevent the release of anticancer drug into the gastrointestinal tract and maintain a high dose concentration to the liver. Thereafter, the vascular sheath was removed using an exchange wire (Fixed core wire guide; Cook Medical, Bloomington, IN, USA), and a 5-Fr Chemo-port catheter was inserted. Two or three side holes were then made at 0.5- or 1-cm intervals from the tip, which was positioned in the hepatic artery proper or in the proximal portion of the gastroduodenal artery, and digital subtraction angiography through the chemo-port catheter was performed to identify the vessels where the chemotherapeutic agent will be infused. Following this, the chemo-port chamber and catheter were connected using connection rings, and the port chamber was implanted into the subcutaneous fat layer below the groin. The function of the chemo-port system was then rechecked using heparinized saline, and position change or catheter kinking was confirmed with fluoroscopy. In the presence of a large extrahepatic tumoral feeder, bland embolization using 150–250 or 355–500 μm of polyvinyl alcohol particles (Contour; Boston Scientific, Marlborough, MA, USA) was performed prior to HAIC implantation.

### Statistics

The aims of this single-arm study were to evaluate the safety and AEs, and to reveal minimal efficacy for the next phase 2 clinical trial; thus, the sample size was not determined based on the statistical power. All statistical analyses were performed using the SPSS software (ver. 13.0; SPSS, Inc., Chicago, IL, USA) and Prism (Ver. 9.0; GraphPad Software, San Diego, CA, USA), and all data were summarized using descriptive statistics. Overall survival (OS) was measured from the date of enrollment to the date of death or the last follow-up visit. Progression free survival was defined as the time from the date of enrollment to the date of disease progression, death, or the last follow-up visit. The Kaplan-Meier method was used to analyze PFS and OS.

## Results

### Patient Characteristics

From March 2016 to June 2021, a total of 11 patients with locally advanced HCC were enrolled into this trial to receive VAX-NK/HCC. Baseline clinical characteristics of all patients are presented in **Table 1**. The median age was 56.6 years (range: 43–71), and a majority of patients were male (90.9%). Most patients were rated as Child-Pugh A (81.1%). However, the similar proportions of the patients were classified as BCLC stage B and C of 54.5% and 45.5%, respectively. Regarding HCC characteristics, eight patients (72.7%) had a multinodular type while three patients (27.3%) had an infiltrative type. Moreover, seven patients (63.7%) had a large tumor exceeding 5 cm in diameter. Eight (72.8%) and five (45.5%) patients had more than five tumors and macrovascular invasion, respectively.

**Table 1:** Baseline clinical characteristics of patients (*n* = 11).

Characteristics	No. of patients (%)
Age, median, years (range)	56.6 (43–71)
Gender	
Male	10 (90.9)
Female	1 (9.1)
ECOG performance status	
0	2 (18.2)
1	9 (81.8)
Child-Pugh class	
A	9 (81.8)
B	2 (18.2)
BCLC stage	
B	6 (54.5)
C	5 (45.5)
Tumor size	
<5 cm	4 (36.4)
5–10 cm	4 (36.4)
≥ 10 cm	3 (27.3)
Number of tumors	
2-5	3 (27.3)
≥ 5	5 (45.5)
≥ 10	3 (27.3)
Macrovascular invasion	
Yes	5 (45.5)
No	6 (54.5)
Etiology	
HBV/HCV/unknown	6 (54.5)/3 (27.3)/2 (18.2)
Extra-hepatic spread	
present/absent	0 (0.0)/11 (100)
Prior therapies	
Surgery plus TACE	4 (36.4)
Surgery plus RT	1 (9.1)
TACE plus sorafenib	1 (9.1)
TACE alone	3 (27.3)
No	2 (18.2)
Morphology of HCC	
Multinodular	8 (72.7)
Infiltrative	3 (27.3)
α-fetoprotein	
≥ 400 μg/L	6 (54.5)
< 400 μg/L	5 (45.5)

BCLC, Barcelona clinic liver cancer; ECOG, Eastern Cooperative Oncology Group; HCC, hepatocellular carcinoma; HBV, hepatitis B virus; HCV, hepatitis C virus; TACE, transarterial chemoembolization; RT, radiotherapy.

### Phenotypic Characteristics and Function of VAX-NK/HCC

The leukapheresis was performed to obtain VAX-NK/HCC after the 3^rd^ HAIC cycle while patients were on 5-FU and cisplatin. The purity, surface markers, and cytotoxicity of VAX-NK/HCC manufactured from 11 enrolled patients are shown in **Table 2**. The purity of expanded NK cells (CD3^-^CD56^+^) markedly increased after culture with the mean value of 81.3%. On the contrary, the proportions of T cell (CD3^+^CD56^-^), NKT cell (CD3^+^CD56^+^), and others (CD3^-^CD56^-^) were as low as 7%, 5%, and 3%, respectively. Expanded NK cells also displayed the high levels of cell surface markers such as CD16, CD69, CD94, and NKG2D with the mean values of 98.4%, 88.8%, 98.1%, and 98.1%, respectively. The functional activity of VAX-NK/HCC was also investigated by *in vitro* cytotoxicity test against K562. VAX-NK/HCC exhibited the strong cytotoxic activity at a 4:1 effector-to-target cell ratio with minimum potency of 70%. Taken together, this data suggest that VAX-NK/HCC manufactured from HCC patients on chemotherapy was highly purified and activated NK cell population.

**Table 2:** Characteristics of VAX-NK/HCC.

Patient no.	Purity (%)			CD3^-^CD56^-^ (others)	Cell Surface Markers (%)				Cytotoxicity (%)
	CD3^-^CD56^+^ (NK cells)	CD3^+^CD56^-^ (T cells)	CD3^+^CD56^+ ^(NKT cells)		CD16^+^	CD69^+^	CD94^+^	NKG2D^+^	
					CD3^-^CD56^+^ (NK cells)				
1	90.4	6.4	2.4	0.6	99.2	79.2	98.3	94.2	74
2	90.0	6.6	1.4	1.7	96.5	81.4	98.1	99.1	79
3	89.8	6.0	2.1	1.9	92.7	83.3	98.8	97.2	70
4	89.5	5.5	2.1	2.6	98.9	94.3	96.4	100	74
5	89.3	6.3	2.4	1.8	99.9	97.3	98.8	90.8	80
6	90.2	5.7	2.1	1.8	98.2	92.3	98.3	99.2	78
7	92.6	4.1	3.0	0.2	99.6	95.0	99.6	100	90
8	91.7	5.4	2.5	0.3	99.6	97.1	99.6	100	84
9	96.9	1.4	1.3	0.1	99.9	82.2	93.3	100	85
10	90.7	6.6	1.7	0.8	98.4	94.3	99.5	99.4	82
11	91.1	5.4	1.7	1.7	99.3	79.9	98.5	99.3	94

NK, natural killer; NKT, natural killer T.

### Safety Assessment

Hematologic and non-hematologic AEs were summarized in **Table 3**. Of the hematologic AEs reported, anemia was the most common, with five patients (45.5%) of any grade and one patient (9.1%) of grade 3 or higher. Moreover, two patients (18.2%) had neutropenia, with one patient of grade 3 and 4 each, both of which recovered successfully upon adequate treatment with granulocyte colony-stimulating factor. Additionally, one patient (9.1%) had grade 2 thrombocytopenia. Serum chemistry dysfunctions were also reported, including four patients (36.4%) with combined hyperkalemia, one (9.1%) with grade 3 hyperkalemia, one with grade 2 hypomagnesemia, and one with grade 2 increased creatinin. On the other hand, non-hematologic AEs of any grade included nausea, fatigue, and rhinorrhea in seven (63.6%), three (27.3%), and two (18.2%) patients, respectively. Ascites, pain, headache, myalgia, vomiting and dizziness were also reported, each occurring in one patient. Of note, fatigue of grade 3 or higher was found in two patients (18.2%), but it was manageable. All adverse events observed in this study were regarded as HAIC therapy-related by the investigators with no AEs related to locoregional NK cell infusion. Furthermore, there were no cases of decompensation or uncontrolled AEs during HAIC treatment. Thus, the treatment was generally well-tolerated regardless of the dose of VAX-NK/HCC administered, with no incidences of grade 3 or 4 AEs attributable to VAX-NK/HCC cell infusion.

**Table 3:** Adverse events during HAIC and VAX-NK/HCC therapy.

A. Hematologic adverse events		
Adverse events	Any Gradenumber (%)	Grade ≥ 3number (%)
Anemia	5 (45.5)	1 (9.1)
Hyperkalemia	4 (36.4)	1 (9.1)
Neutropenia	2 (18.2)	2 (18.2)
Thrombocytopenia	1 (9.1)	0 (0)
Increased creatinine	1 (9.1)	0 (0)
Hypomagnesemia	1 (9.1)	0 (0)
B. Non-hematologic adverse events		
Adverse events	Any Gradenumber (%)	Grade ≥ 3number (%)
Nausea	7 (63.6)	0 (0)
Fatigue	3 (27.3)	2 (18.2)
Rhinorrhea	2 (18.2)	0 (0)
Ascites	1 (9.1)	0 (0)
Pain	1 (9.1)	0 (0)
Headache	1 (9.1)	0 (0)
Myalgia	1 (9.1)	0 (0)
Vomiting	1 (9.1)	0 (0)
Dizziness	1 (9.1)	0 (0)

HAIC, hepatic arterial infusion chemotherapy; HCC, hepatocellular carcinoma; NK, natural killer.

### Efficacy

Of the 13 patients who had received 2 cycles of 5-FU and cisplatin-based HAIC as initial therapy, 11 patients showing SD or better received 3 doses of VAX-NK/HCC, and all were evaluable for the treatment response. The best responses are shown in **Table 4**. The objective response rate (ORR) was 63.6%, including four CR (36.4%) and three PRs (27.3%). In addition, SD was observed in two patients (18.2%), and PD in two patients (18.2%), resulting in the DCR of 81.8%.

**Table 4:** Therapeutic response after HAIC and VAX-NK/HCC therapy (*n* = 11).

	Number of patients (%)
Best overall response	
Complete response	4 (36.4)
Partial response	3 (27.3)
Stable disease	2 (18.2)
Progressive disease	2 (18.2)
Objective response rate	7 (63.6)
Disease control rate	9 (81.8)

HAIC, hepatic arterial infusion chemotherapy; HCC, hepatocellular carcinoma; NK, natural killer.

**Figure 2:**
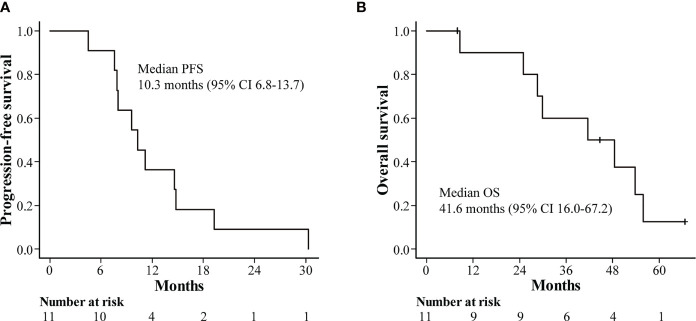
Kaplan-Meier estimates of survival outcomes. **(A)** progression-free survival. **(B)** overall survival. CI, confidence interval.

**Figure 3:**
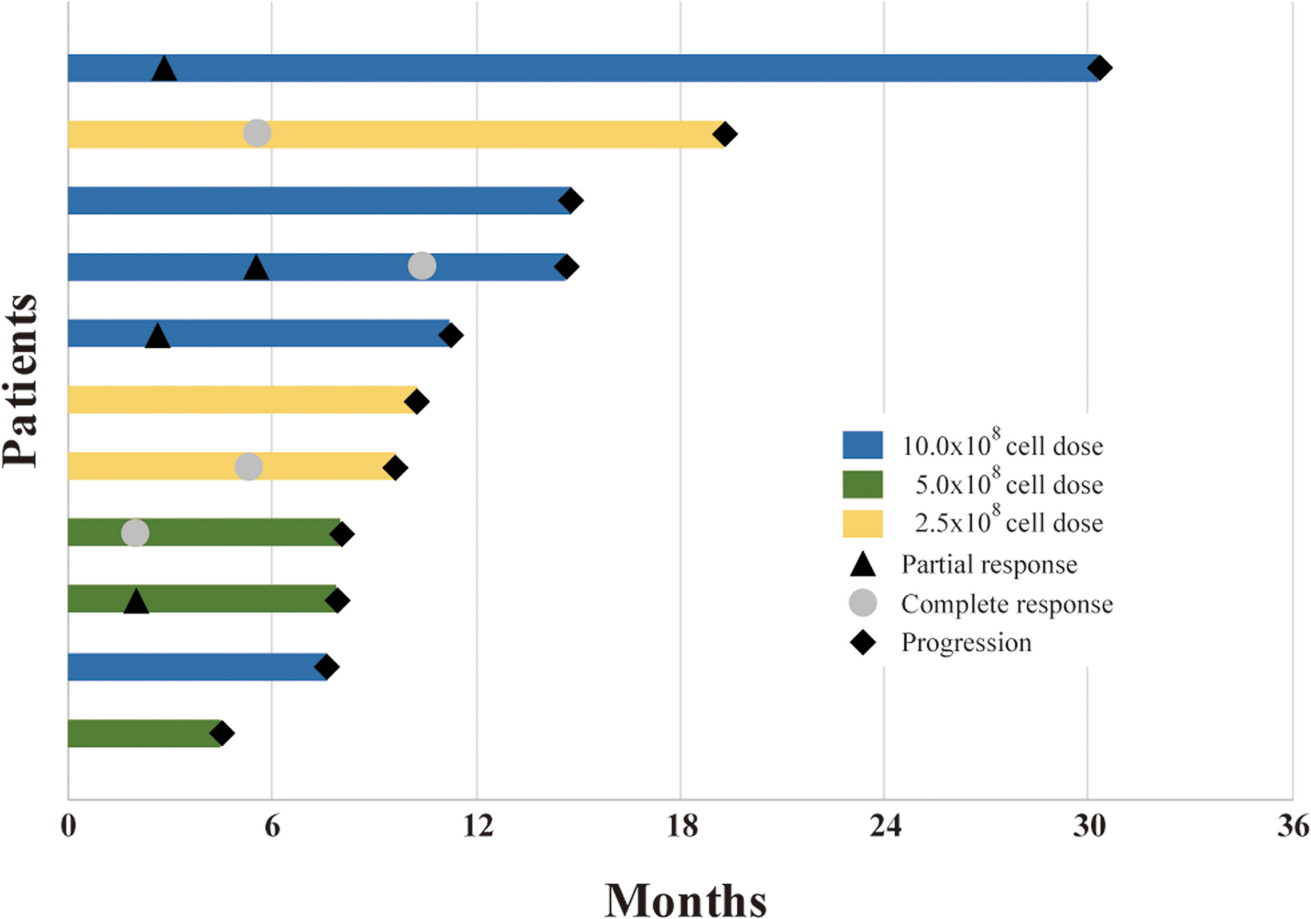
Time to progression and duration of response in patients with an objective response.

The median follow-up time in this study was 55.9 months (range: 44.7–63.4 months). The median PFS in all patients was 10.3 months (95% confidence interval [CI]: 6.8–13.7), with PFS rates of 36.4% and 9.1% at 12 and 24 months, respectively (**Figure 2A**). The median OS was 41.6 months (95% CI: 16.0–67.2), with OS rates of 72.7% and 54.5% at 12 and 36 months, respectively (**Figure 2B**). Furthermore, the median duration of response (DOR) was reported to be 8.6 months among seven patients showing a complete response or partial response according to mRECIST (range: 4.3–27.5 months) (**Figure 3**).

**Figure 4:**
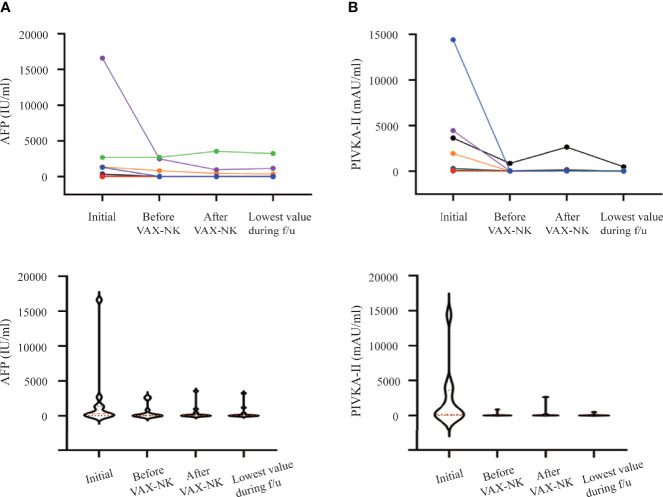
Changes in tumor marker levels during and after treatment. **(A)** α-fetoprotein (AFP) (IU/mL). **(B)** protein-induced by vitamin K absence or antagonist-II (PIVKA-II) (mAU/mL). NK, natural killer.

Two serological biomarkers of HCC were measured to monitor the progression of the disease. The α-fetoprotein (AFP) and protein-induced by vitamin K absence or antagonist-II (PIVKA-II) levels were detected from the initial screening to the last follow-up (**Figure 4**). The median AFP level of 305.8 IU/ml at initial screening was decreased to 15.4 IU/ml following HAIC and VAX-NK/HCC administration (**Figure 4A**). Likewise, the post-treatment median PIVKA-II level was also decreased to 49.0 mAU/ml from 182.0 mAU/ml at initial screening (**Figure 4B**).

### Immunological Response

**Figure 5:**
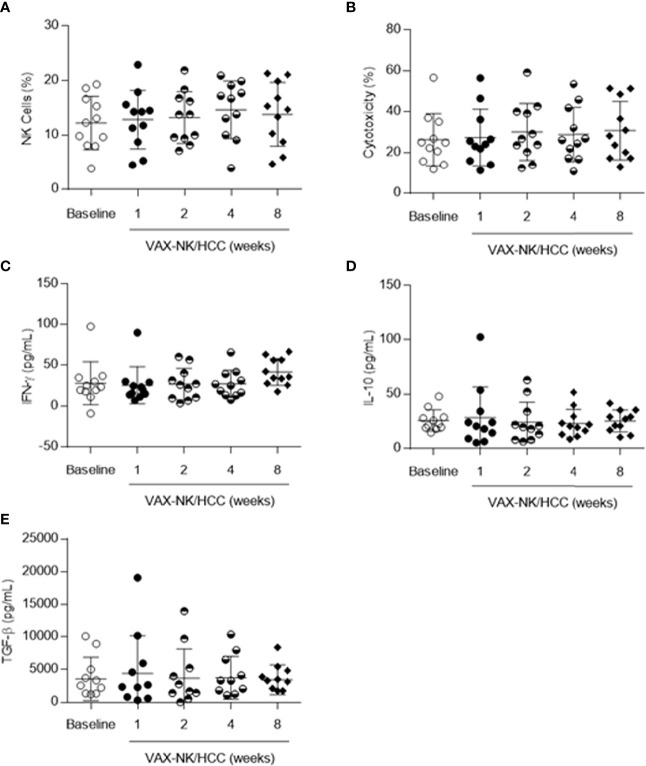
Immunoprofiling during and after treatment. **(A)** Percentages of peripheral NK cells with CD3^-^CD56^+^ before and after locoregional NK cell infusion. **(B)** The cytotoxic activity of PBMCs against K-562 cells at effector to a 10:1 E:T ratio. **(C–E)** The serum cytokine levels of IFN-γ, IL-10, and TGF-β.

The Immunological profiling was performed for all patients using PBMCs at the baseline, and 1, 2, 4, and 8 weeks after the locoregional infusion of VAX-NK/HCC into the liver (**Figure 5** & **Supplementary Figure 1**). The proportion of NK cells in the peripheral blood was measured up to approximately 20% in all patients, regardless of the cell numbers infused (**Figure 5A**). There was a slight increase in the blood NK cell proportion after VAX-NK/HCC infusion in five patients, but it was not significant compared to the level at the baseline. Similarly, CD4/CD8 T cell and lymphocyte/monocyte ratios were not significantly different before and after NK cell infusion in the peripheral blood (data not shown). The cytotoxic activity of PBMCs against K562 cells was also tested, and it was found to be similar ranging from 10% nearly up to 60% at both the baseline and post-treatment time points (**Figure 5B**). Of interest, a slight increase in cytotoxicity was also observed in two patients with elevated levels of peripheral NK cells. As last, the serum cytokine levels of IFN-γ, IL-10, and TGF-β were analyzed, and there was no significant change after treatment (**Figures 5C–E**).

## Discussion

The present study demonstrates the safety and clinical activity of locoregional high-dose NK cell therapy combined with HAIC in patients with locally advanced HCC. The combined treatment of NK cells with HAIC of 5-FU and cisplatin was well tolerated with no unexpected toxicities. To the best of our knowledge, this is the first clinical study of NK cell therapy combined with HAIC, the liver locoregional therapy against locally advanced HCC. The clinical responses from this treatment were also promising with the ORR of 63.6%, and the median PFS and OS of 10.3 and 41.6 months, respectively, despite most of these patients receiving prior therapies.

Sorafenib is the recommended first-line treatment in advanced HCC (2). Notably, the SHARP trial has shown that among advanced HCC patients, the sorafenib group had a nearly 3-month median survival benefit in comparison to the placebo group (10.7 *vs*. 7.9 months; P < 0.001) (3). In the sub-analyses of the SHARP trial, however, sorafenib did not show a definitely prolonged OS among the BCLC B subgroup in which patients with intermediate or locally advanced HCC belong to (12). In addition, the clinical effect of sorafenib becomes less evident in hepatitis B endemic areas. For example, the median OS and TTP of sorafenib group was 6.5 and 2.8 months, respectively as compared to 4.2 and 1.4 months in the placebo group, respectively in another randomized controlled trial from the Asia and Pacific region (9). Therefore, the limitations of sorafenib, such as its modest efficacy, adverse effects, and high cost, have made it difficult to use in clinical practice in this intermediate and/or locally advanced HCC (13). Given the heterogeneity of advanced HCC population, a multi-modal treatment strategy has been suggested for the successful treatment of advanced stage of HCC.

NK cells are cytotoxic innate lymphocytes that have regulatory functions against viral infections and tumors, acting as key anti-tumor effectors in the human immune system (14). In particular, studies have shown decreases in peripheral blood NK cells among patients with HCC, suggesting that the dysfunction or exhaustion of NK cells might contribute to HCC development and progression (4, 15–18). Thus, several NK cell-based immunotherapies against HCC have been explored both at the pre-clinical and clinical levels against HCC. In this study, NK cells were administered locoregionally *via* the hepatic artery following four cycles of HAIC. It is assumed that systemic administration of NK cells would be disadvantageous for reaching the target organs, considering the tumor microenvironment (TME) in which cytolytic effectors including NK and CD8^+^ T cells have low capacity to infiltrate (19). In this regard, the local injection of NK cells *via* the hepatic artery may be an intriguing approach to improve the NK cell homing and infiltration to solid tumors, as it directly delivers viable NK cells to the target organ, which is the liver in this case. Furthermore, it has been reported that 5-FU increased NK cell activity while decreasing myeloid-derived suppressor cells and regulatory T cells, resulting in the possibility of a favorable immune response in HCC (20, 21). In addition, cisplatin can enhance the efficacy of NK cell-based immunotherapy by up-regulating UL16-binding protein 2 (ULBP2), a NKG2D ligand, on HCC (22). Therefore, this would provide the study rationale that the pretreatment with 5-FU and cisplatin-based cytotoxic chemotherapy would be helpful in overcoming the immune suppressive TME and enhancing the anti-tumor activity of NK cells synergistically.

Although HAIC has yet to become a standard treatment for advanced HCC, there have been several reports showing the positive efficacy and survival benefit of HAIC in intermediate and/or locally advanced HCCs (23–27). Song et al. reported a comparative study between sorafenib and HAIC in advanced HCC with portal vein tumor thrombus (PVTT) in which HAIC showed significantly longer OS (7.1 *vs*. 5.5 months, *p* = 0.011) and a favorable treatment response with ORR (24% *vs*. 13.3%, *p* = 0.214) and DCR (90% *vs*. 45%, *p* < 0.001) compared to sorafenib (28). Other studies have also supported the favorable response of HAIC in locally advanced HCCs regardless of PVTT (29, 30). This accumulating evidence led to the conclusion that HAIC could be a potential front-line treatment choice in a subpopulation without extrahepatic metastasis (25, 31). Given these findings, it is reasonable to speculate that locoregional high-dose NK cell therapy with HAIC may further improve the clinical outcome in intermediate and/or locally advanced HCCs as compared to systemic therapies, such as sorafenib. Supportive of this notion, the efficacy of locoregional high-dose NK cell therapy with HAIC in this trial was promising in advanced HCC patients with macrovascular invasion and no extrahepatic metastasis in that it clearly showed a better median PFS (10.3 months, 95% CI: 6.8–13.7) and median OS (41.6 months, 95% CI: 22.5–60.7) as compared to those of the HAIC therapy alone from previous studies whose median OS ranged from 7.3 to 14.0 months, and median TTP or PFS ranged from 2.0 to 7.0 months (23–27). Although this was not a comparative study, these results suggest that NK cell infusion after HAIC had a positive effect against locally advanced HCCs. Although several adverse reactions occurred during treatment, most of them were related to conventional chemotherapeutic agents in HAIC. There were no cases of grade 3 or 4 adverse events related to VAX-NK/HCC cell infusion at any dose. Furthermore, the patterns of adverse reactions in this study were similar to those of HAIC from previous studies in HCC (24, 32), suggesting that VAX-NK/HCC has a favorable safety profile in this study population.

Despite these findings, there were certain limitations in this study. First, we were unable to verify the immunologic response of VAX-NK/HCC, which might be due to the small number of enrolled patients and the possibility of loco-regional delivery and retention in the liver of NK cells infused. Thus, a large cohort study will be necessary to determine its immunologic response. Second, after the combined VAX-NK/HCC and HAIC treatment, progression or recurrence occurred over time in all cases of this study. This study included only four cycles of HAIC and 5 consecutive day infusion of VAX-NK/HCC infusion. In our opinion, HAIC or VAX-NK/HCC infusion should be continued to maintain their therapeutic responses.

## Conclusion

Our results demonstrate that the combination of HAIC and locoregional high-dose NK cell therapy is a safe and effective treatment for locally advanced HCC patients who were refractory to the standard treatment. However, as there are limited clinical data available, further comparative studies between HAIC with VAX-NK/HCC and HAIC alone, or between HAIC with VAX-NK/HCC and sorafenib will be required to confirm the study findings.

## Data Availability Statement

The raw data supporting the conclusions of this article will be made available by the authors, without undue reservation.

## Ethics Statement

The studies involving human participants were reviewed and approved by Chonnam National University Hwasun Hospital. The patients/participants provided their written informed consent to participate in this study.

## Author Contributions

WB, BL, SC, and YK conceived and designed the study. HK and IC collected the data. WB, HJK, YK, and JL analyzed and interpreted the data. All authors were involved in the drafting, review, and approval of the report and the decision to submit for publication.

## Funding

This work was supported by a National Research Foundation of Korea (NRF) grant (No. 2020R1A5A2031185), and by the Bio & Medical Technology Development Program of the NRF funded by the Ministry of Science and ICT (MSIT) (No. NRF-2017M3A9E2056372 and No. NRF-2020M3A9G3080281). 

## Conflict of Interest

JL is employed by Vaxcell-Bio Therapeutics.

The remaining authors declare that the research was conducted in the absence of any commercial or financial relationships that could be construed as a potential conflict of interest.

## Publisher’s Note

All claims expressed in this article are solely those of the authors and do not necessarily represent those of their affiliated organizations, or those of the publisher, the editors and the reviewers. Any product that may be evaluated in this article, or claim that may be made by its manufacturer, is not guaranteed or endorsed by the publisher.

## Supplementary Material

Supplementary Figure 1 Immunoprofiling during and after treatment. Graphs in Figure 5 are represented as in line and dot plot format. **(A)** Percentages of peripheral NK cells with CD3^-^CD56^+^ before and after locoregional NK cell infusion. **(B)** The cytotoxic activity of PBMCs against K-562 cells at effector to a 10:1 E:T ratio. **(C–E)** The serum cytokine levels of IFN-γ, IL-10, and TGF-β.
