# Supplementary figures and images for: A Phase I Study of Locoregional High-Dose Autologous Natural Killer Cell Therapy With Hepatic Arterial Infusion Chemotherapy in Patients With Locally Advanced Hepatocellular Carcinoma
Source: Front Immunol. 2022 Jun 2;13:879452. doi: 10.3389/fimmu.2022.879452 (PMC9202498; doi:10.3389/fimmu.2022.879452)

**A**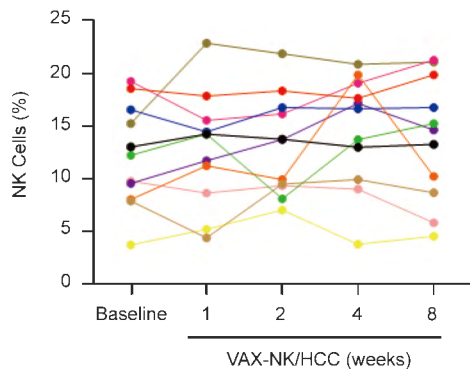**B**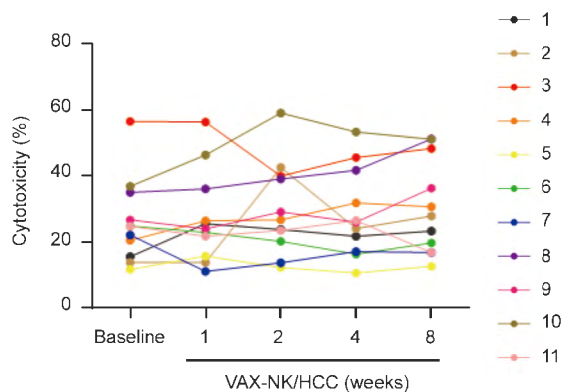**C**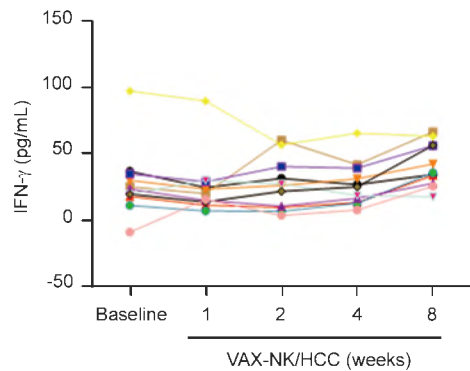**D**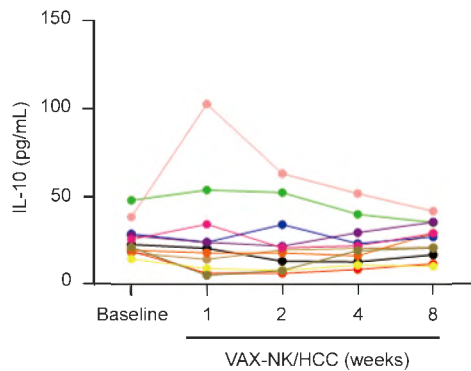**E**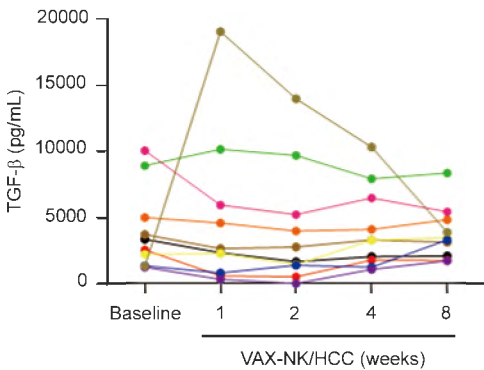

Supplement: Supplementary Figure 1 — Immunoprofiling during and after treatment. Graphs in Figure 5 are represented as in line and dot plot format. (A) Percentages of peripheral NK cells with CD3-CD56+ before and after locoregional NK cell infusion. (B) The cytotoxic activity of PBMCs against K-562 cells at effector to a 10:1 E:T ratio. (C–E) The serum cytokine levels of IFN-γ, IL-10, and TGF-β. [file DataSheet_1.pdf]
